# Supplementary material for: An inorganic mineral-based protocell with prebiotic radiation fitness
Source: Nat Commun. 2023 Dec 5;14:7699. doi: 10.1038/s41467-023-43272-5 (PMC10698201; doi:10.1038/s41467-023-43272-5)
Supplement: Supplementary file 1 — Supplementary Information [file 41467_2023_43272_MOESM1_ESM.pdf]

Supplementary Information

An inorganic mineral-based protocell with prebiotic radiation fitness

Shang Dai[1,2#], Zhenming Xie[1#], Binqiang Wang[1#], Rui Ye[3#], Xinwen Ou[3], Chen Wang[4], Ning Yu[1], Cheng Huang[1], Jie Zhao[1], Chunhui Cai[1], Furong Zhang[1], Damiano Buratto[1,3], Taimoor Khan[1,3], Yan Qiao[5]*, Yuejin Hua[1,6,7]*, Ruhong Zhou[1,2,3,7]*, Bing Tian[1,7]*

1. Institute of Biophysics, College of Life Sciences, Zhejiang University, Hangzhou, China; 2. Shanghai Institute for Advanced Study of Zhejiang University, Shanghai, China; 3. School of Physics, Institute of Quantitative Biology, Zhejiang University, Hangzhou, China; 4. College of Pharmaceutical Science, Zhejiang University, Hangzhou, China; 5. Institute of Chemistry, Chinese Academy of Sciences, Beijing, China; 6. Qian Xuesen Collaborative Research Center of Astrochemistry and Space Life Sciences, Ningbo University, Ningbo, China; 7. Cancer Center, Zhejiang University, Hangzhou, China

# These authors contributed equally to this work.

*Correspondence: tianbing@zju.edu.cn; rhzhou@zju.edu.cn; yjhua@zju.edu.cn; yanqiao@iccas.ac.cn

Supplementary Note

Description of the peptide screening for polyP-peptide coacervate microdroplet formation

We screened various positively charged amino acids and short peptides for their potential in coacervate microdroplet formation with polyP. We mixed 100 mM positively charged amino acids (Arginine, Lysine, and Histidine), 20 mM dipeptides (RR, KK, HH) or tripeptides (RRR, KKK, HHH, RER, ERR, and RRE) with 0.2 mM polyP (n=100) solution in equal volume at room temperature, respectively. The results show that the positively charged amino acid alone could not form coacervate microdroplet with polyP, while the dipeptides and tripeptides formed microdroplets with polyP (Supplementary Fig. 1b). Among them, the tripeptides (RER, ERR, RRE) have better performance in coacervate microdroplet formation. The RER were selected in our following experiments based on following considerations: First, the better performance in coacervate microdroplet formation, and amino acid sequence in the tripeptide has not substantial effect on microdroplet formation; Second, arginine (R) is the major component of histone for DNA binding in eukaryotic cell chromatin; Glutamic acid (E), an oxygen donor amino acid, was proposed to be interacted with manganese ion, which can form into antioxidant Mn-peptides [1].

Supplementary Methods

Preparation of coacervate microdroplets

The coacervate microdroplets dispersions were prepared by mixing equal volume aqueous solution of anionic polyP and cationic metal salt/oligopeptides at room temperature. Typically, 0.5 mM polyP solution was mixed with 50 mM metal salt solution; 0.2 mM polyP solution was mixed with 100 mM amino acid solution or 20 mM oligopeptide solution.

Zeta potential of coacervate microdroplets

Zeta potential analysis was performed using a Malvern Zetasizer Nano-ZS instrument equipped with a 633nm laser[2]. Samples were injected into a disposable zeta cuvette at 25°C with scattering angle of 175°.

Raman spectra

2.5 μL of 10 mM RER solution was dripped on the detection chip (Hooke Instruments, Changchun, China). Then the samples were analyzed using a HOOKE P300 confocal Raman spectrometer (Hooke Instruments, Changchun, China). For sample analysis, the acquisition time was set to 10 s and the laser power at the sample was 5.0 mW[3]. All Raman spectra were processed with background subtraction and baseline correction using HOOKE INTP software (v 1.0) (Hooke Instruments, Changchun, China).

Effects of external salts and metal chelate on coacervate microdroplets

To investigate the effects of external salts and metal chelate on coacervate microdroplets, we exposed the coacervates to 0.1-0.6 M NaCl and 20 M EDTA solutions (final concentration), respectively. The preparation of polyP coacervates was followed as described above. The polyP Mn coacervates was prepared by mixing equal volume of 0.5 mM polyP with 50 mM $MnCl_2$, and the polyP-RER coacervates was prepared by mixing equal volume of 0.2 mM polyP with 20 mM RER. After adding equal volume of NaCl or EDTA solution to coacervate microdroplets suspension, morphology of the coacervate microdroplets was observed under a Nikon ECLIPSE Ti2-U Inverted fluorescence microscope (Nikon Co., Japan).

Recruitment process of biomolecules by coacervate microdroplets

To evaluate the sequestration of ssDNA, dsDNA and protein by coacervate microdroplets, the

freshly prepared coacervate microdroplets were incubated with FAM-ssDNA (green fluorescent), FAM-dsDNA and mCherry protein (red fluorescent), respectively. Briefly, 10μL coacervate microdroplets was incubated with 2 μL FAM-ssDNA solution or FAM-dsDNA (10 μM), and 2 μL mCherry solution (0.5 mg/mL) at room temperature for 5 min. Then, the samples were imaged under Nikon ECLIPSE Ti2-U Inverted Fluorescence Microscope without further treatment. The images were analyzed using NIS-Elements D software (v4.60.00).

Coacervate microdroplet fusion

The fusion tests between the coacervates microdroplets in aqueous solution were conducted by mixing the prepared polyP coacervate microdroplets at equal volume ratio. The fusion processes of the polyP-Mn, and polyP-RER coacervate microdroplets were monitored under bright field mode of a Nikon fluorescence microscope respectively. The fusion processes of the mCherry and FAM-ssDNA sequestered polyP-RER coacervate microdroplets were monitored using fluorescent microscopy mode of the Nikon fluorescence microscope respectively. Images are representative of at least three independent replicates.

Fluorescence recovery after photobleaching (FRAP)

FRAP was performed on a Leica THUNDER Imager Live Cell confocal laser scanning microscope with a 100× oil objectives. The mCherry was excited at 514 nm, and emission signals were collected in the range 585-635nm. Ten images at attenuated laser intensity (20% intensity) were taken before photobleaching. Photobleaching was performed using a single pulse of argon laser (405 nm) at 50% intensity for mCherry (25 frames) through a round region of interest (ROI) of nominal diameter of 1.0-3.0 μm. The laser was then switched back to attenuated intensity and the recovery images were recorded for 74 seconds (500 frames). The time interval of each frame is 0.148s. The FRAP data

were analyzed using Leica Application Suite X (LAS X) software (v3.3.0.16799).

SDS-PAGE analysis of *β*-galactosidase

SDS-PAGE was performed to detect the *β*-galactosidase in supernatant and inside of protocells. Protocells collected by centrifugation were added with 5 μL 5 x SDS loading buffer. The supernatant and protocells were then subjected to 12% SDS-PAGE. *β*-galactosidase solution was used as the control.

EPR spectroscopy measurements

For the hydroxyl radical scavenging assay using EPR spectroscopy, Mn-antioxidant complexes with small molecules used in hydroxyl radical scavenging measurement were prepared as described in Supplementary Table 1. Samples for the measurement of hydroxyl radicals were added with free radical trapping agent (DMPO, final concentration 200 mM)[4]. When all the samples were ready for next step, the prepared samples were given 1 kGy γ-ray radiation dose. After irradiation, the samples were stored on dry ice for subsequent EPR measurement.

EPR spectroscopy measurement of free Mn^{2+} in protocell were performed following the method as described[5]. The protocell sample used for EPR analysis was prepared as described before, and 1 mM $MnCl_2$ solution was used as control.

100 μL of all the samples were transferred into a capillary glass tube, and were in turn introduced into a quartz tube. The recording of the EPR spectra of the samples was initiated at room temperature with an X-band EPR spectrometer (Bruker ESRA-300, Germany). The instrument conditions were the following: frequency of approximately 9.8 GHz with 100 kHz modulation, central field ± sweep width of 3514 G ± 60 G (for DMPO-$^{\bullet}$OH assay); 3000G ± 3000 G (for Mn^{2+} assay), microwave power of 20 mW, time constant of 81.92 ms, sweep time 40 s. The obtained EPR spectra were

analyzed using Bruker WinEPR Processing software (v2.22Rev.12) (Bruker Co.).

Urea-PAGE assay of polyP under γ-ray radiation

Urea-PAGE assay of polyP solution were performed following the method as described previously[6].

Molybdenum blue assays of Pi release

Pi release of polyP solution treated with different γ-ray doses were monitored using molybdenum blue assay[7] with modifications. Molybdenum blue is a blue compound whose absorbance at 660 nm shows a positive correlation with the concentration of free Pi. Using the molybdenum blue spectrophotometry method, the concentration of free Pi in the sample solution can be measured. The polyP solution samples were treated with 0-1 kGy γ-ray dose (Institute of Crops and Nuclear Technology, Academy of Agricultural Sciences, Zhejiang, China). For the Pi release of protocell, the protocell sample was lysed by 20 μL 100 mM EDTA solution, then the lysed samples were diluted to 500 μL using deionized water. Then, 200 μL of 50 g/L ammonium molybdate sulfuric acid (sulfuric acid concentration: 2M) solution, 100 μL sodium sulfite solution (200g/L) and 100 μL hydroquinone solution (5g/L) were added into 500 μL sample solution.

After incubating at room temperature for 30 minutes, the reaction product was measured at 660 nm. The released Pi content was calculated by substituting the value measured by A_{660} into the standard curve.

^{31}P NMR spectra

^{31}P NMR assays of polyP solution were performed following the method as described previously[8]. Briefly, 0.5 mg polyP was dissolved in 1 mL heavy water and treated with different doses of γ-ray irradiation. After radiation, the samples were analyzed by Bruker AVANCE NEO nuclear magnetic resonance spectrometer. The scanning time of the NMR spectrometer was 2 hours and the scanning

frequency was 400MHz. The NMR spectra were analyzed using MestReNova software (v11.0.4-18998) (Mestrelab Research S.L.). The peaks at approximately 0, -5 to -10, and -20 to -22 ppm represent the Q^0 (Orthophosphates), Q^1 (End of chain phosphorus atoms) and Q^2-middle (Middle of chain phosphorus atoms in polyphosphates), respectively [8].

Electrospray ionization-mass spectrometry (ESI-MS)

Electrospray ionization mass spectrometry (ESI-MS) were performed using AB Sciex Triple TOF 5600+ spectrometer (AB Sciex, Framingham, USA), equipped with a DuoSpray™ Turbo V electrospray ionization source. Spectrum were recorded in the positive ion modes in the range 100 to 2000 m/z. The instrumental parameters were as follows: scan range m/z 100–2000, dry gas–nitrogen, temperature 170 °C, ion energy 5 eV [1].

Protocell sample was prepared as described above. Specifically, 100 μL polyP-Mn coacervate suspension was mixed with 100 μL polyP-RER coacervate suspension. After incubating for 5 min at room temperature, the mixture was centrifuged for 2 min at 8000 rpm. The supernatant was removed, and the precipitation was resuspended with 200 μL deionized water with ultrasonic treatment conducted for 5 min to make it fully dissolved. The resuspended protocell solution was directly loaded into ESI-MS for analysis. Data were processed using Peakview software (v1.2) (AB Sciex, Framingham, USA).

$O_2^{\bullet -}$ scavenging assay

$O_2^{\bullet -}$ scavenging activities of Mn-antioxidants were measured using the Total Superoxide Dismutase Assay Kit with WST-8 (Beyotime Co, China). The Mn-antioxidants were prepared using the method as described previously with modification[9]. In this study, Mn-antioxidant complexes with small molecules were prepared as shown in the following Supplementary Table 1.

Supplementary Table 1. Composition of Mn-antioxidant complexes with small molecules

	Mn-Pi	Mn-RER	Mn-RER-Pi
10mM $MnCl_2$	10μL	10μL	10μL
10mM RER		10μL	10μL
10mM PiB	10μL		10μL
ddH_2O	80μL	80μL	70μL
Total volume	100μL	100μL	100μL

*PiB: phosphate buffer pH 7, containing 10mM Na_3PO_4 (pH 8) and 10mM NaH_2PO_4 (pH 6)

20 μL of the Mn-antioxidant complex mixture was used for subsequent determination of superoxide anion scavenging activity, and deionized water was used as a control. The absorbance of the reaction product was measured at 450 nm. Relative superoxide anion scavenging percentage = $(A_{control} - A_{sample}) / (A_{control} - A_{blank}) \times 100\%$.

Supplementary Figures

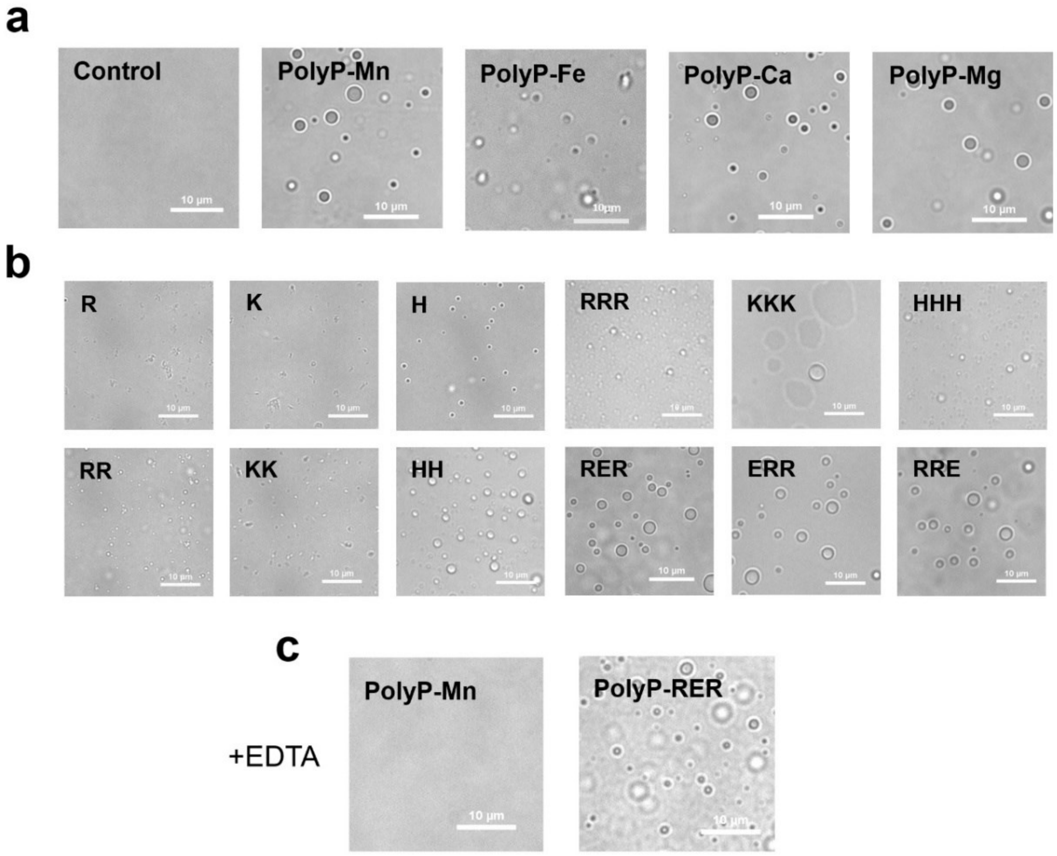

Supplementary Fig. 1. Effect of divalent metal cations, positively charged amino acids and oligopeptides on the formation of polyP-based coacervate microdroplets. (**a**) Bright field image of coacervate microdroplet formation by mixing 0.2 mM polyP (n=100) solution with 100 mM divalent metal cation solution ($MnCl_2$, $FeCl_2$, $CaCl_2$, $MgCl_2$) in equal volume at room temperature, respectively. Scale bar, 10 μm. Control, 0.2 mM polyP (n=100) solution; (**b**) Bright field image of coacervate microdroplet formation by mixing 0.2 mM polyP (n=100) solution with 100 mM amino acid (Arginine, Lysine, and Histidine) or 20 mM peptide (RR, KK, HH, RRR, KKK, HHH, RER, ERR and RRE) solution in equal volume at room temperature, respectively. Scale bar, 10 μm. (**c**) Bright field image of polyP-Mn and polyP-RER coacervate microdroplet exposed to 20 mM EDTA solution at room temperature, respectively. Scale bar, 10 μm. Images in this figure represented the results of three independently repeated experiments. Source images are provided as a Source Data file.

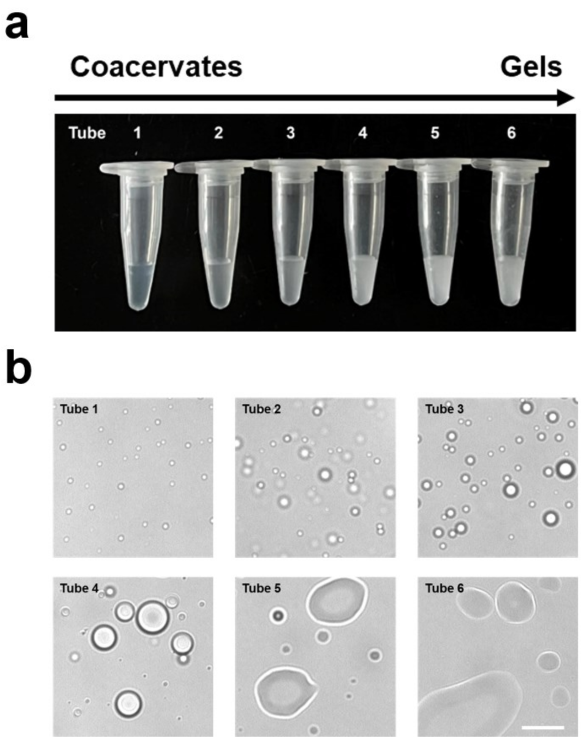

Supplementary Fig. 2. Concentration-dependent morphology of polyP-Mn coacervate microdroplets and polyP-Mn gel-like material in centrifuge tube (a) and microscopic field (b). Tube 1, 0.2 mM polyP and 10 mM $MnCl_2$; Tube 2, 0.5 mM polyP and 50 mM $MnCl_2$; Tube 3, 1 mM polyP and 75 mM $MnCl_2$; Tube 4, 2.5 mM polyP and 200 mM $MnCl_2$; Tube 5, 5 mM polyP and 500 mM $MnCl_2$; Tube 6, 10 mM polyP and 1 M $MnCl_2$. Scale bar, 10 μm. Experiments were independently repeated three times with similar results. Source images are provided as a Source Data file.

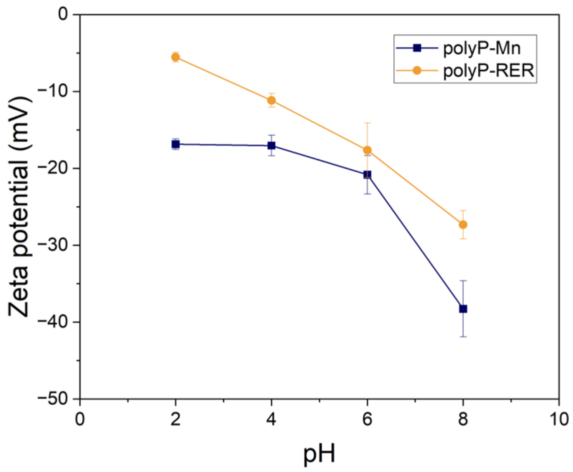

Supplementary Fig. 3. Zeta potential of polyP-based coacervate microdroplets at different pH. Data presented as mean ± SD (n=3). Source data are provided as a Source Data file.

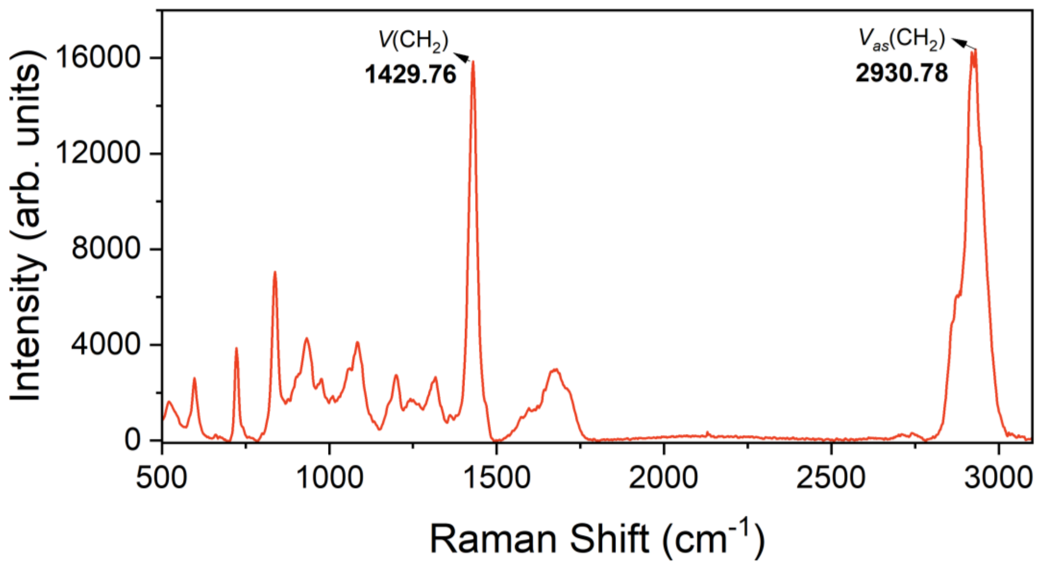

Supplementary Fig. 4. Raman spectra of RER (10 mM). The Raman spectra showed stretching vibration of symmetrical CH_2 and asymmetric CH_2 bonds at 1429.76cm^{-1} and 2930.78cm^{-1} in RER solution. The a.u. indicate arbitrary units. Source data are provided as a Source Data file.

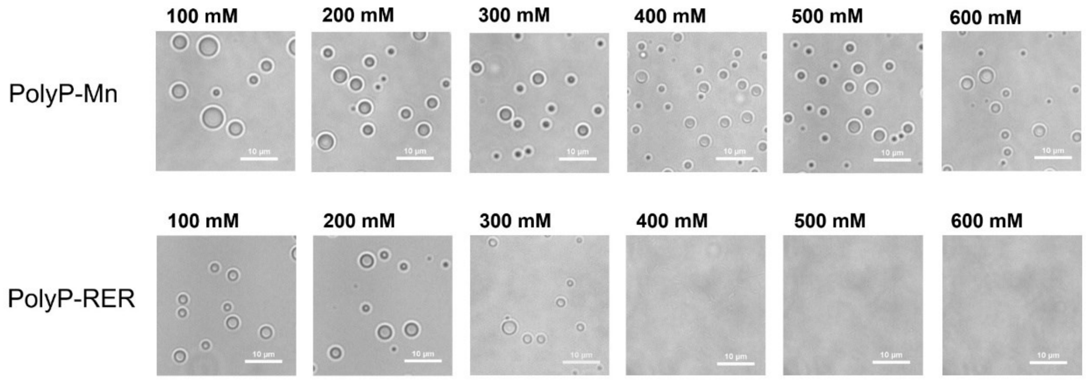

Supplementary Fig. 5. Effect of NaCl on the coacervate microdroplets. Bright field image of polyP-Mn and polyP-RER coacervate microdroplet exposed to 0.1-0.6 M NaCl solution at room temperature, respectively. Scale bar, 10 μm. Images in this figure represented the results of three independently repeated experiments. Source images are provided as a Source Data file.

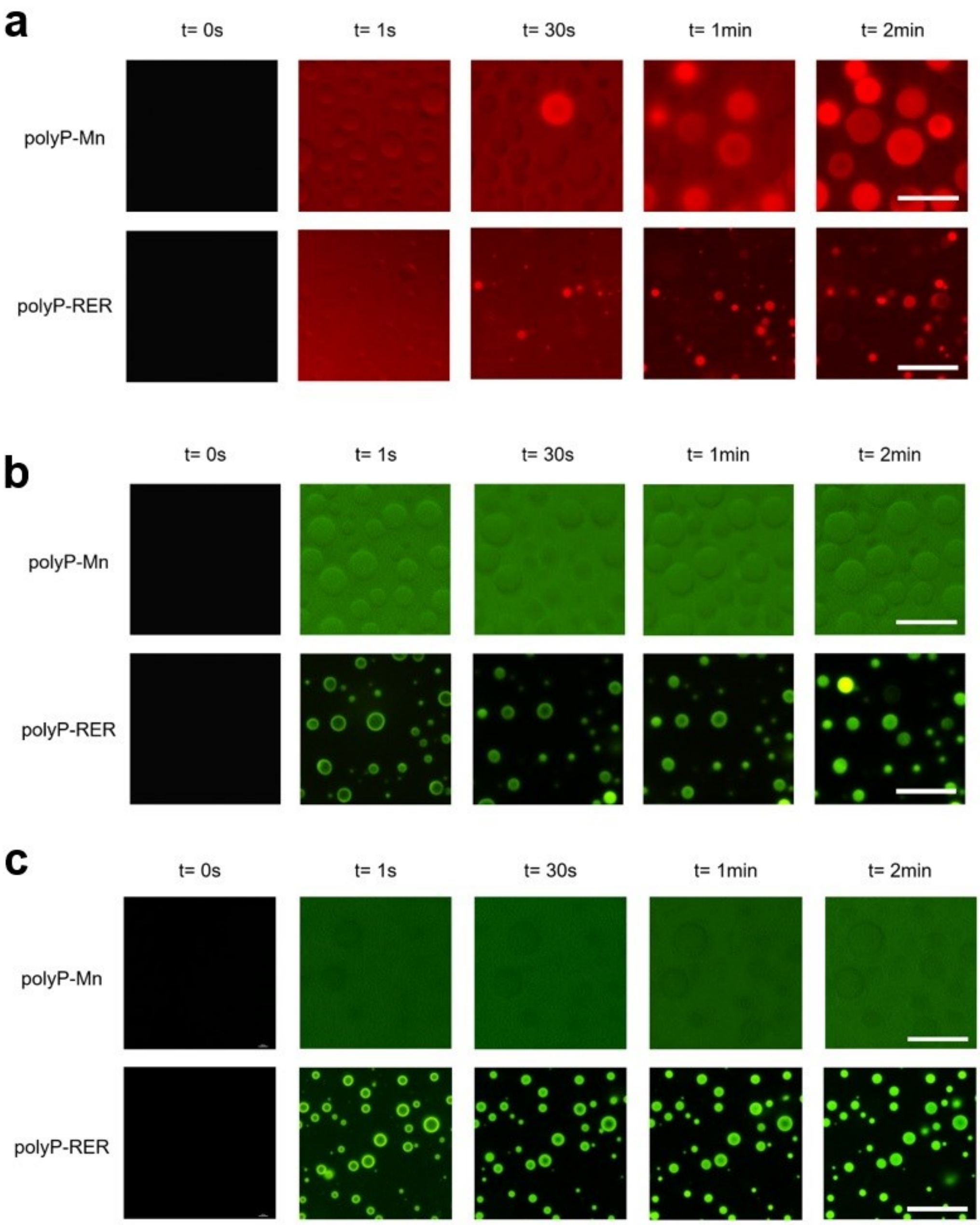

Supplementary Fig. 6. Recruitment process of biomacromolecules in the two type of coacervate microdroplets. (**a**) Time course of mCherry recruitment by polyP-Mn and polyP-RER coacervate microdroplets, respectively. (**b**) Time course of FAM-ssDNA recruitment by polyP-Mn and polyP-RER coacervate microdroplets, respectively. Scale bar: 5 μm. (**c**) Time course of FAM-dsDNA recruitment by polyP-Mn and polyP-RER coacervate microdroplets, respectively. Scale bar: 5 μm. Images in this figure represented the results of three independently repeated experiments. Source images are provided as a Source Data file.

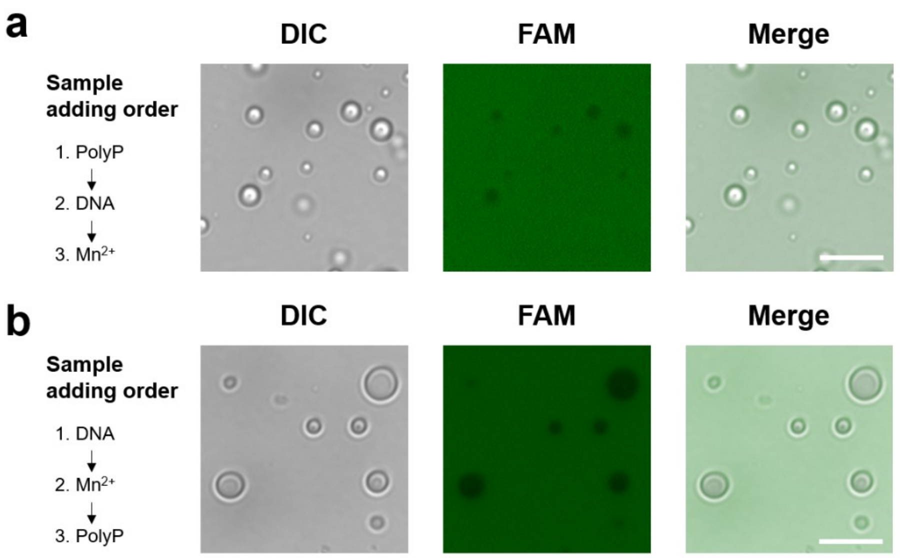

Supplementary Fig. 7. Effect of the sample adding order on recruitment of DNA (FAM-ssDNA, green fluorescent) by microdroplets. Incubation conditions are as described in the Methods section. The sample adding order is indicated by arrow: 1**.** polyP, 2. DNA, 3. Mn^{2+} in (**a**); 1. DNA, 2. Mn^{2+}, 3. polyP in (**b**). Scale bar: 10 μm. Images in this figure represented the results of three independently repeated experiments. Source images are provided as a Source Data file.

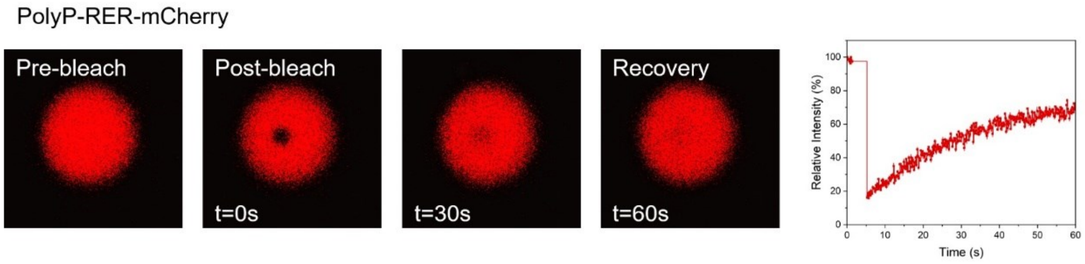

Supplementary Fig. 8. FRAP assays of polyP-RER-mCherry coacervate microdroplets. Fluorescence recovery after photobleaching (left panels) and FRAP recovery curve of the coacervate microdroplets (right panel) were shown. Experiments were independently repeated three times with similar results. Source data are provided as a Source Data file.

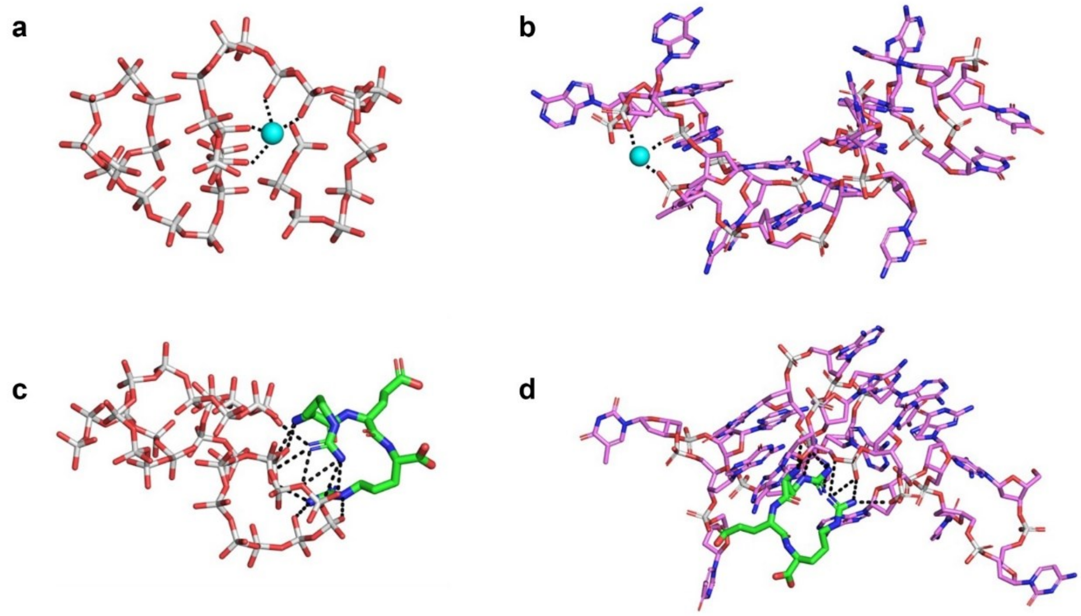

Supplementary Fig. 9. Representative snapshots of Mn^{2+} ion binding to polyP (a), Mn^{2+} ion binding to ssDNA (b), tripeptide RER binding to polyP (c), and RER binding to ssDNA (d). The PolyP chain, Mn^{2+} ion, ssDNA and RER chain are colored in white, cyan, purple and green, respectively. For clarity, the phosphorus atoms, oxygen atoms, and nitrogen atoms are specifically shown in white, red, and blue, respectively.

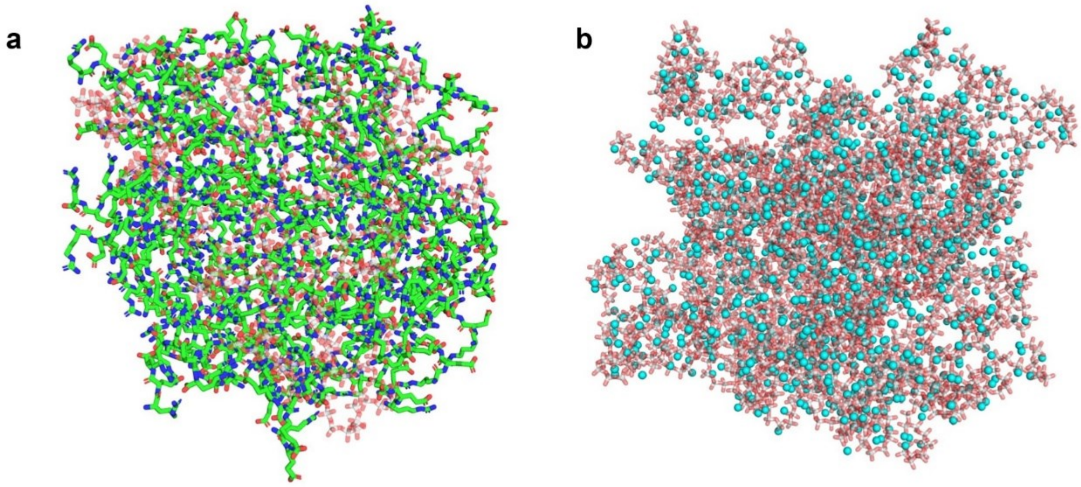

Supplementary Fig. 10. The final conformations of the polyP-RER mixed cluster (a) and the polyP-Mn^{2+} mixed cluster (b). The polyP chain, Mn^{2+} ion, and RER chain are colored in white, cyan and green, respectively. Phosphorus atoms, oxygen atoms, and nitrogen atoms are specifically shown in white, red, and blue, respectively. The polyP is shown transparently.

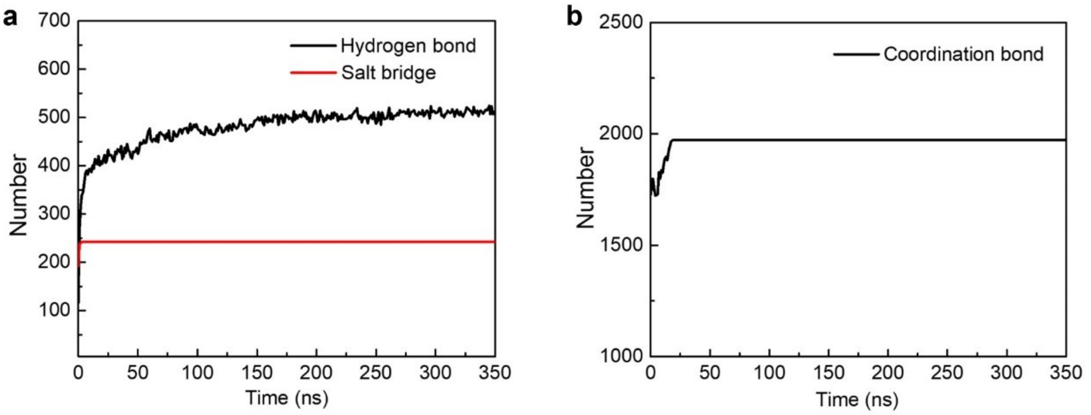

Supplementary Fig. 11. The number of hydrogen bonds and salt bridges between polyP and RER in the polyP-RER mixed cluster (a), and the number of coordinate bonds between polyP and Mn^{2+} ion in the polyP-Mn^{2+} mixed cluster (b).

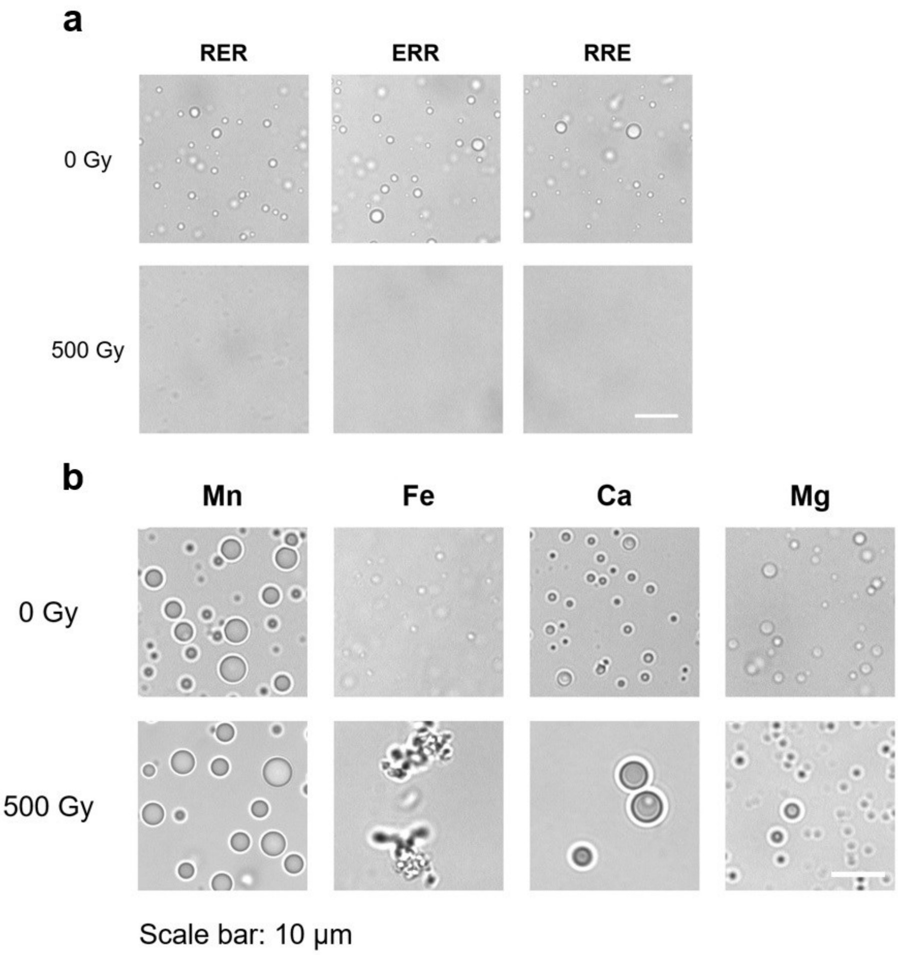

Supplementary Fig. 12. Bright field image of polyP-RER (a) and polyP-metal (b) microdroplets exposed to 500 Gy irradiation at room temperature, respectively. Images in this figure represented the results of three independently repeated experiments. Source images are provided as a Source Data file.

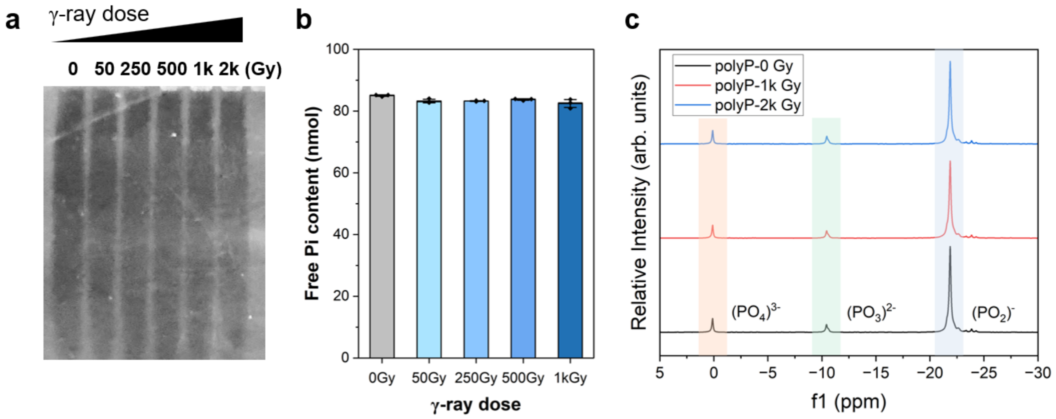

Supplementary Fig. 13. Urea-PAGE, Pi release and ^{31}P NMR measurement of polyP solution irradiated using γ-ray at different doses. (**a**) Urea-PAGE analysis of polyP under the treatment with γ-ray at different doses (0, 50, 250, 500, 1 k and 2 kGy). Experiments were independently repeated three times with similar results. (**b**) Molybdenum blue assay of free phosphate (Pi) release from polyP solution under the treatment with γ-ray at different doses (0, 50, 250, 500 and 1 kGy). Data presented as mean ± SD (n=3); (**c**) ^{31}P NMR spectra of polyP solution under the treatment with γ-ray at different doses (0, 1 and 2 kGy). Source data are provided as a Source Data file.

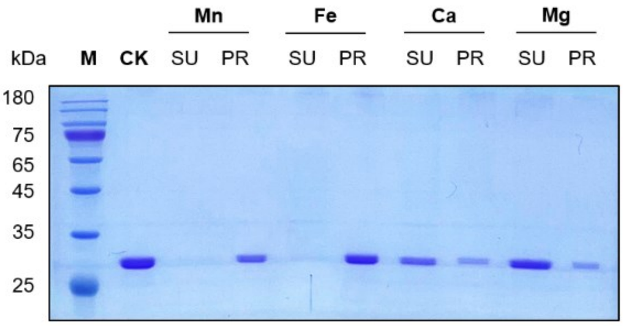

Supplementary Fig. 14. SDS-PAGE analysis of recruitment of mCherry by polyP-metal coacervate droplets. M, protein marker; CK, mCherry; SU: supernatant of the coacervate droplet solution separated by centrifugation; PR: precipitate (coacervate droplets) prepared from the coacervate droplet solution by centrifugation. Experiments were independently repeated three times with similar results. Uncropped gel image is provided as a Source Data file.

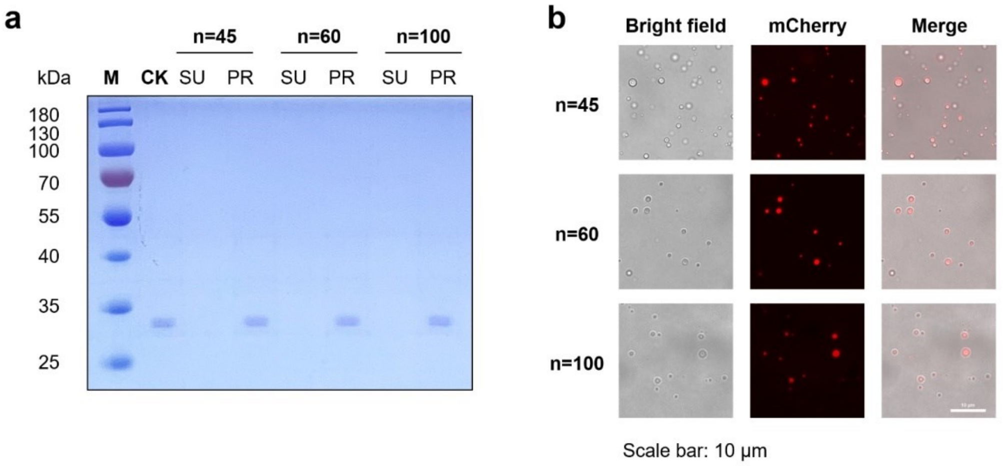

Supplementary Fig. 15. Recruitment of mCherry by polyP (n=45, 60 or 100)-Mn coacervate droplets. (**a**) SDS-PAGE analysis of the recruited mCherry in droplets. M, protein marker; CK, mCherry; SU: supernatant of the coacervate droplet solution separated by centrifugation; PR: precipitate (coacervate droplets) prepared from the coacervate droplet solution by centrifugation. Experiments were independently repeated three times with similar results. Uncropped gel image is provided as a Source Data file. (**b**) Microscope images of polyP (n=45, 60 or 100)-Mn coacervate droplets containing mCherry, respectively. Images in **b** represented the results of three independently repeated experiments. Source images are provided as a Source Data file.

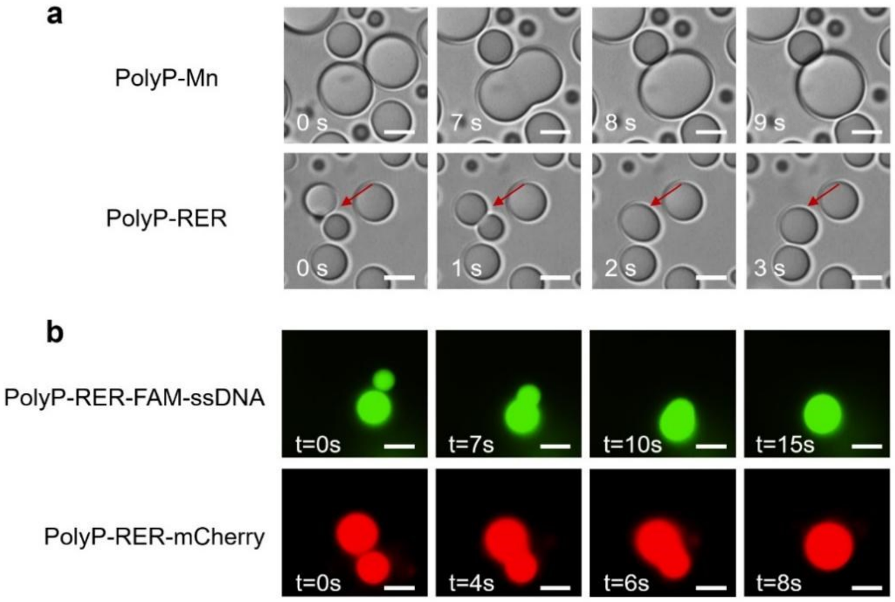

Supplementary Fig. 16. Fluidity and fusion properties of polyP based coacervate microdroplets. (**a**) Time course of droplet fusion of polyP-Mn and polyP-RER, respectively. Scale bar: 2 μm; (**b**) Time course of droplet fusion of polyP-RER coacervate microdroplets sequestered with FAM-ssDNA or mCherry protein. Scale bar: 2 μm. Images in this figure represented the results of three independently repeated experiments. Source images are provided as a Source Data file.

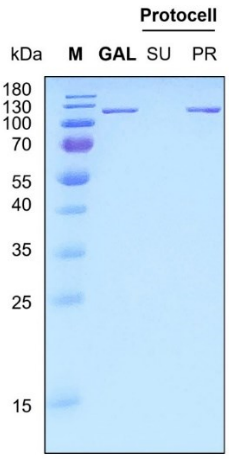

Supplementary Fig. 17. Recruitment ability of coacervate droplets on *β*-galactosidase. SDS-PAGE analysis of the recruited *β*-galactosidase in protocell. M, protein marker; GAL, *β*-galactosidase; SU: supernatant of the coacervate droplet solution separated by centrifugation; PR: precipitate (coacervate droplets) prepared from the coacervate droplet solution by centrifugation. Experiments were independently repeated three times with similar results. Source image is provided as a Source Data file.

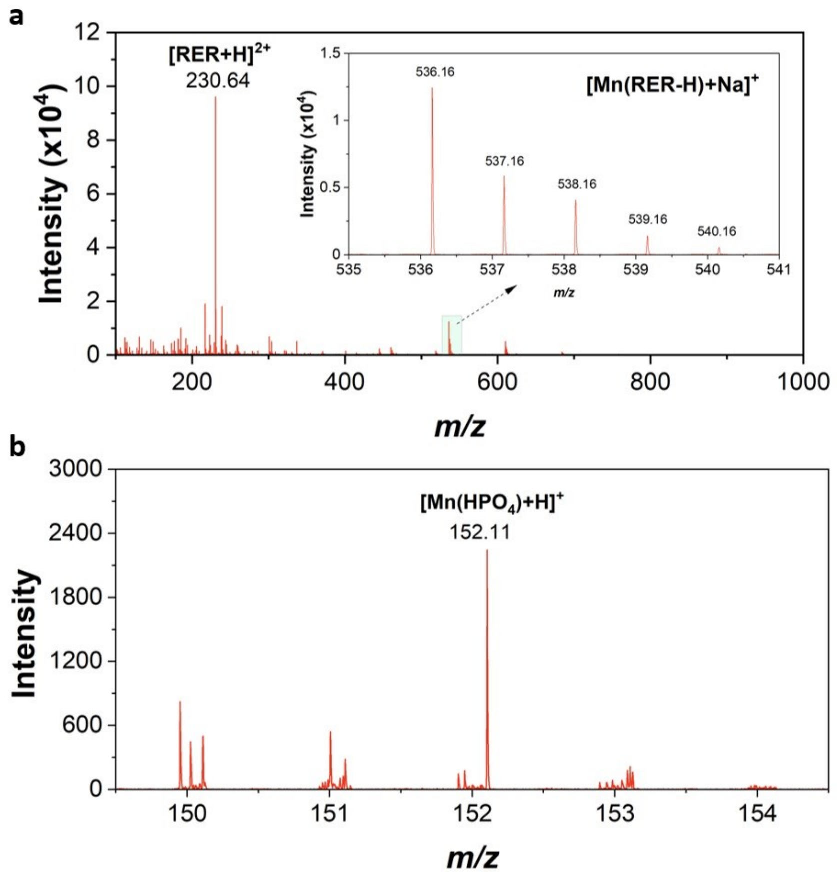

Supplementary Fig. 18. ESI-MS spectra of Mn complexes with small molecules in protocell. (**a**) $[Mn(RER\text{-}H)+Na]^+$ (*m/z* = 536.16) and $[RER+H]^{2+}$ (*m/z* = 230.64) was corresponding to Mn-RER and free RER, respectively. (**b**) $[Mn(HPO_4)+H]^+$ (*m/z* =152.11) might be corresponding to Mn-Pi. Source data are provided as a Source Data file.

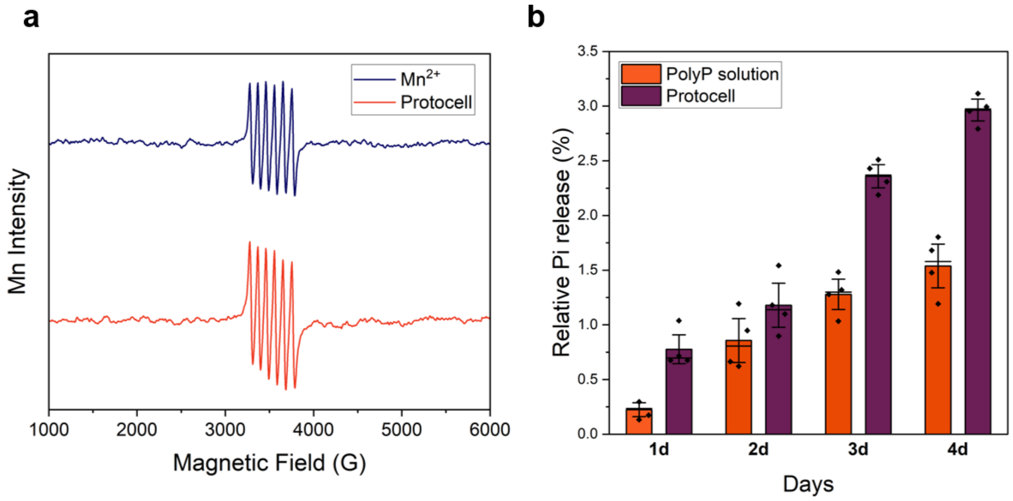

Supplementary Fig. 19. Free Mn^{2+} in protocells and phosphate (Pi) release in the protocells. (**a**) EPR assays of Mn^{2+} in protocells exposed to γ-ray at 1 kGy. 1 mM manganese chloride was used as control; (**b**) Relative Pi release fraction (%) in protocells stored at 37°C for different times (after 1, 2, 3 and 4 days). PolyP solution was used as control. Data presented as mean ± SD (n = 4). Source data are provided as a Source Data file.

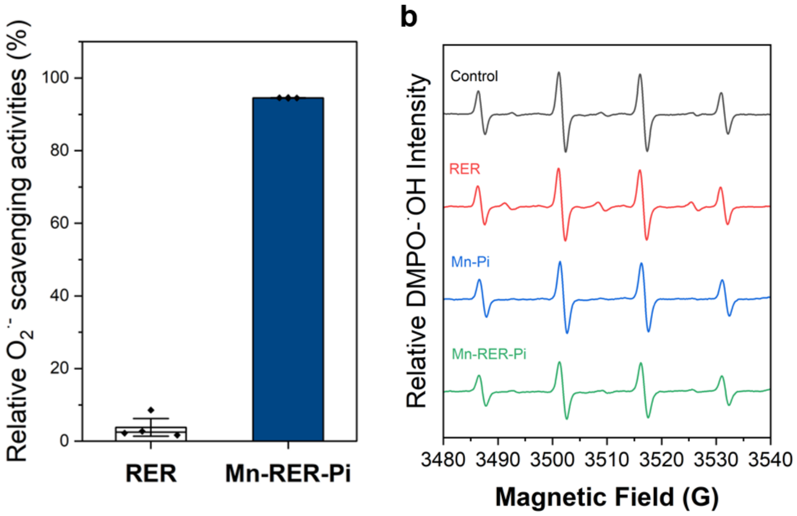

Supplementary Fig. 20. ROS scavenging activity of Mn-antioxidant complexes. (**a**) Relative $O_2^{\bullet-}$ scavenging activity of various Mn-antioxidant complexes in solution. Data presented as mean ± SD(n=4). Deionized water was used as the control; (**b**) EPR spectra of the DMPO-•OH intensity of various Mn-antioxidant complexes in solution treated with 1 kGy γ-ray radiation. Deionized water was used as control. Source data are provided as a Source Data file.

Source data for gel images:

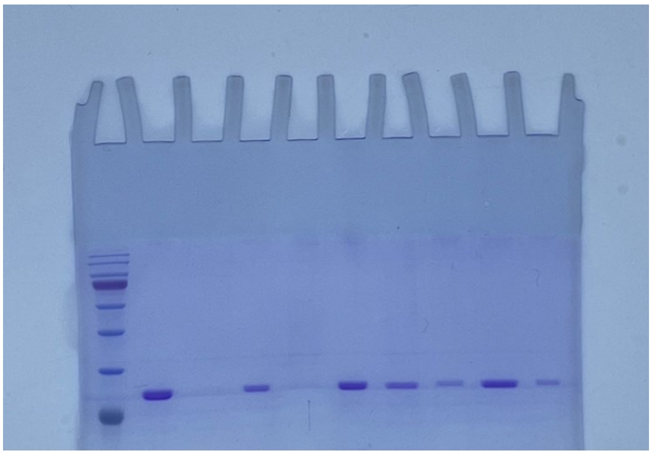

Supplementary Fig. 14. SDS-PAGE analysis of recruitment of mCherry by polyP-metal coacervate droplets.

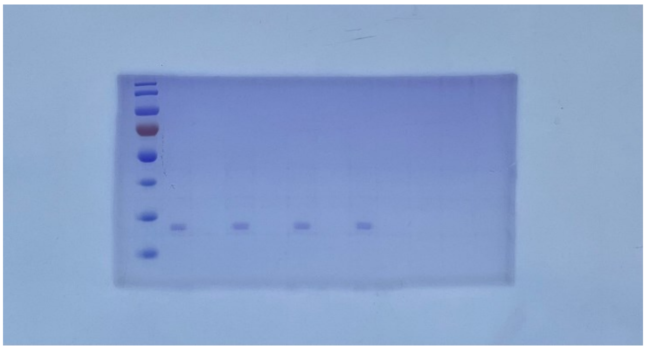

Supplementary Fig. 15. Recruitment of mCherry by polyP (n=45, 60 or 100)-Mn coacervate droplets. (**a**) SDS-PAGE analysis of the recruited mCherry in droplets.

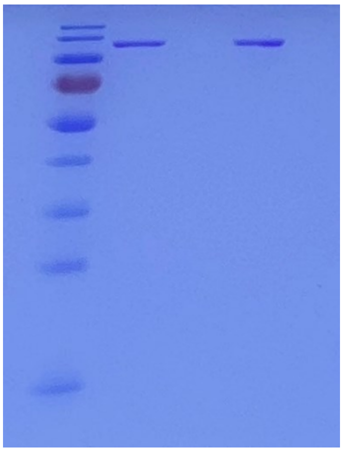

Supplementary Fig. 17. Recruitment ability of coacervate droplets on *β*-galactosidase.

Supplementary References

1. Peana, M. *et al.* Manganese binding to antioxidant peptides involved in extreme radiation

resistance in *Deinococcus radiodurans*. *J. Inorg. Biochem.* **164**, 49–58 (2016).

2. Zhang, Y. *et al.* Giant Coacervate Vesicles As an Integrated Approach to Cytomimetic Modeling. *J. Am. Chem. Soc.* **143**, 2866–2874 (2021).

3. Wang, D. *et al.* Intracellular polyphosphate length characterization in polyphosphate accumulating microorganisms (PAOs): Implications in PAO phenotypic diversity and enhanced biological phosphorus removal performance. *Water Res.* **206**, 117726 (2021).

4. Zhang, L. *et al.* Knockout of crtB or crtI gene blocks the carotenoid biosynthetic pathway in *Deinococcus radiodurans* R1 and influences its resistance to oxidative DNA-damaging agents due to change of free radicals scavenging ability. *Arch. Microbiol.* **188**, 411–419 (2007).

5. Bruch, E. M., de Groot, A., Un, S. & Tabares, L. C. The effect of gamma-ray irradiation on the Mn(II) speciation in *Deinococcus radiodurans* and the potential role of Mn(II)-orthophosphates. *Metallomics* **7**, 908–916 (2015).

6. Dai, S. *et al.* Dynamic Polyphosphate Metabolism Coordinating with Manganese Ions Defends against Oxidative Stress in the Extreme Bacterium *Deinococcus radiodurans. Appl. Environ. Microbiol.* **87**, (2021).

7. Holman, W. I. M. A new technique for the determination of phosphorus by the molybdenum blue method. *Biochem. J.* **37**, 256–259 (1943).

8. Momeni, A. Degradation and hemostatic properties of polyphosphate coacervates. *Acta Biomater.* **14** (2016).

9. Daly, M. J. *et al.* Small-Molecule Antioxidant Proteome-Shields in *Deinococcus radiodurans*. *PLoS ONE* **5**, e12570 (2010).
