# Supplementary material for: An inorganic mineral-based protocell with prebiotic radiation fitness
Source: Nat Commun. 2023 Dec 5;14:7699. doi: 10.1038/s41467-023-43272-5 (PMC10698201; doi:10.1038/s41467-023-43272-5)
Supplement: Supplementary file 3 — Description of Additional Supplementary Files [file 41467_2023_43272_MOESM3_ESM.pdf]

# Description of Additional Supplementary Files

**File name: Supplementary movie**

Description: Time course of spontaneous fusion of polyP-Mn droplets with polyP-RER-DNA droplets.
